# Extended Right Hepatectomy following Clearance of the Left Liver Lobe and Portal Vein Embolization for Curatively Intended Treatment of Extensive Bilobar Colorectal Liver Metastases: A Single-Center Case Series

**DOI:** 10.3390/curroncol31030085

**Published:** 2024-02-21

**Authors:** Sebastian Knitter, Linda Sauer, Karl-H. Hillebrandt, Simon Moosburner, Uli Fehrenbach, Timo A. Auer, Nathanael Raschzok, Georg Lurje, Felix Krenzien, Johann Pratschke, Wenzel Schöning

**Affiliations:** 1 Department of Surgery, Campus Charité Mitte and Campus Virchow-Klinikum, Charité—Universitätsmedizin Berlin, Corporate Member of Freie Universität Berlin and Humboldt-Universität zu Berlin, 13353 Berlin, Germany; 2 Department of Radiology, Charité—Universitätsmedizin Berlin, Corporate Member of Freie Universität Berlin and Humboldt-Universität zu Berlin, 13353 Berlin, Germany

**Keywords:** liver surgery, two-staged hepatectomy, colorectal liver metastases, extended right hepatectomy

## Abstract

Background: Two-staged hepatectomy (TSH) including portal vein embolization (PVE) may offer surgical treatment for extensive bilobar colorectal liver metastases (CRLM). This study aimed to investigate the feasibility and outcomes of extended right hepatectomy (ERH) within TSH including PVE for patients with extended CRLM. Methods: We retrospectively collected data of patients who underwent TSH for extended CRLM between 2015 and 2021 at our institution. Clearance of the left liver lobe (clear-up, CU) associated with PVE was followed by ERH. Results: Minimally invasive (*n* = 12, 46%, MIH) or open hepatectomy (*n* = 14, 54%, OH) was performed. Postoperative major morbidity and 90-day mortality were 54% and 0%. Three-year overall survival was 95%. Baseline characteristics, postoperative and long-term outcomes were comparable between MIH and OH. However, hospital stay was significantly shorter after MIH (8 vs. 15 days, *p* = 0.008). Additionally, the need for intraoperative transfusions tended to be lower in the MIH group (17% vs. 50%, *p* = 0.110). Conclusions: ERH following CU and PVE for extended CRLM is feasible and safe in laparoscopic and open approaches. MIH for ERH may result in shorter postoperative hospital stays. Further high-volume, multicenter studies are required to evaluate the potential superiority of MIH.

## 1. Introduction

Colorectal cancer (CRC), among the most prevalent cancer types, is anticipated to account for approximately 1.9 million new cases and 0.9 million cancer-related deaths annually worldwide [1,2]. For patients diagnosed with CRC, liver metastases are present in up to a quarter of cases, and over the course of their disease, up to half of the patients develop metastatic spread to the liver [3,4]. Recent advances in personalized chemotherapy regimen, resection techniques and perioperative management have increased the number of patients eligible for surgical resection of advanced colorectal liver metastases (CRLM), ultimately leading to improved long-term outcomes with five-year survival rates over 50% [5,6].

However, patients with extensive bilobar liver spread and an insufficient future liver remnant (FLR) have traditionally been excluded from curatively intended surgical resections, resulting in a subset where only 10–25% of patients may profit from extensive liver surgery [7]. To address this issue, the concept of two-staged hepatectomy (TSH) was introduced in 2000 by Adam et al., involving a two-step surgical process [8]: First, the FLR is cleared of CRLM in the initial surgery (“clear-up”, CU), which may be combined with local ablation. Afterwards, the contralateral portal vein is embolized to induce hypertrophy of the FLR. After a growth period of approximately 4–8 weeks, a major hepatectomy of the contralateral liver is performed as the second stage [8]. TSH has been established as a standard approach in the multimodal treatment of patients with extensive CRLM, demonstrating promising long-term outcomes with median overall survival of 37 to 50 months, while reporting morbidity rates of 40–47% [9,10]. However, most studies have included standard as well as extended hemi-hepatectomies, and an exclusive analysis of extended right hepatectomies (ERH) after CU and PVE has not been published to date [9,10,11,12].

Therefore, the objective of this single-center case series was to evaluate the safety, feasibility and long-term outcome of ERH after CU and PVE in the context of TSH for patients initially deemed ineligible for resection of CRLM. Specifically, our aims were to analyze short-term postoperative outcomes, including postoperative morbidity and liver surgery-specific complications such as bile leakage, post-hepatectomy liver failure (PHLF), and post-hepatectomy hemorrhage (PHH). Additionally, we aimed to evaluate long-term outcomes by examining overall and disease-free survival following ERH.

## 2. Materials and Methods

### 2.1. Patient Inclusion Criteria and Study Design

Clinicopathological data of all consecutive patients who underwent ERH after CU and PVE for bilobar CRLM at the Department of Surgery, Campus Charité Mitte and Campus Virchow-Klinikum, Charité—Universitätsmedizin Berlin between 2015 and 2021 were, retrospectively, collected. Only patients who received curatively intended treatment, defined as the ability to address all radiologically evident disease, and successfully completed both steps of the procedure, were included in the study. Patients were excluded if they underwent multivisceral resection involving other organs than the liver and the biliary system, underwent the ALPPS (associating liver partition with portal vein ligation for staged hepatectomy) procedure, or were below 18 years of age at the time of resection. Approval for the study was obtained from the institutional ethics commission (EA2/006/16).

### 2.2. Preoperative Evaluation

The routine evaluation of patients included a standardized medical history, physical examination, laboratory tests, and imaging. All patients were discussed in our institutional multidisciplinary tumor board, which consisted of experienced hepatobiliary surgeons, radiologists, oncologists, pathologists and hepatologists [13]. Preoperative chemotherapy, with or without targeting agents based on mutational analysis, was generally administered to all patients [14,15]. Tumor staging and estimation of the FLR were conducted using triphasic contrast-enhanced computed tomography and/or contrast-enhanced magnetic resonance imaging with liver-specific agents [14,15,16]. A recommendation for TSH was made when resection appeared feasible while preserving sufficient vascular supply and biliary drainage, but the FLR was expected to be inadequate for a one-stage approach. Additionally, only patients exhibiting tumor downsizing or at least stable disease after chemotherapy were considered eligible for the procedure. In cases where patients presented with synchronous CRLM, the primary tumor was either resected during CU or subsequently after completing TSH.

### 2.3. TSH and Perioperative Management

Procedures for CU included either atypical or segmental resection, local ablative therapy, or a combination of both. Local ablation was used for lesions <3 cm that were not accessible for parenchymal-sparing resections. In some cases, the primary tumor was resected during the first stage based on the recommendation of the tumor board. PVE was either performed during surgery through catheterization of an ileocolic vein or subsequently via ultrasound-guided transhepatic intervention [17]. Transhepatic PVE procedures were performed by an experienced interventional radiologist using Contour PVA Embolization Particles in combination with Interlock Embolization Coils (Boston Scientific, Marlborough, MA, USA), or Tornado Embolization Coils (Cook Medical, Bloomington, IN, USA). Four weeks after PVE, the grade of hypertrophy was evaluated using computer tomography, and the FLR was calculated. In addition, the LiMAx (maximum liver function capacity) test was performed before CU to assess liver function before and, by interpolating the anticipated value using the calculated FLR, after surgery [18,19,20]. If sufficient liver function was anticipated as measured by the calculated post-surgery LiMAx value, and relevant tumor progression was ruled out, ERH was performed.

As necessitated by tumor spread, ERH was performed in all patients. ERH was defined by the resection of more than 4 continuous liver segments, that is segments 4–8, according to Couinaud’s classification of liver segments [21]. In some cases, segment 1 was also removed if affected by CRLM. Biliary and vasculary reconstruction was performed as necessary. All procedures were performed by five experienced hepatobiliary surgeons. The approach for ERH was either open (OH) or minimally invasive (MIH), including laparoscopic, hand-assisted (HALS) and robotic-assisted approaches, depending on patient-related factors and surgeon’s preference. A history of multiple prior abdominal surgeries did not exclude patients from MIH [22]. For robotic surgery (RS), the DaVinci Xi^®^ Surgical System (Intuitive Surgical Inc., Sunnyvale, CA, USA) was used [23,24]. At first, the abdominal cavity was examined for peritoneal dissemination. Intraoperative ultrasound was routinely used to accurately locate tumors in relation to the hepatic vasculature and biliary system, aiding in defining the transection plane. Dissection of liver parenchyma was conducted using various instruments based on the chosen surgical approach, as previously reported [25].

Following ERH, all patients were admitted to our specialized surgical intensive care unit (ICU) [25]. Histopathological analysis of resected specimens was performed by our institutional pathologists, and negative resection margins (R0) were defined as microscopically absence of tumor cells within 1 mm from the transection plane. Additionally, the presence of fibrosis was classified in grade 0 to 4 according to the Desmet and Scheuer scoring system [26]. All cases were presented in our institutional tumor board to determine further treatment recommendations based on international guidelines [14].

### 2.4. Postoperative Outcomes

Postoperative morbidity within 90 days after surgery was stratified according to the Clavien-Dindo classification [27]. Major morbidity was defined as any complication graded ≥3a. Post-hepatectomy-specific complications such as hemorrhage (post-hepatectomy hemorrhage, PHH) [28], liver failure (post-hepatectomy liver failure, PHLF) [29] and bile leakage [30], were defined based on the International Study Group of Liver Surgery (ISGLS) criteria and categorized into three grades, respectively. Wound infections or intraabdominal abscesses were defined according to the Centers for Disease Control and Prevention (CDC) definition for surgical site infections (SSI) [31].

### 2.5. Statistical Analysis

Patients were stratified into MIH and OH groups based on the surgical approach of ERH and were then compared by clinicopathological parameters. Categorical variables were expressed as totals and frequencies, and continuous variables were presented as medians with ranges. Statistical comparisons were performed using the Fisher’s exact, chi-square, or Mann–Whitney *U* test as appropriate. Overall survival (OS) and disease-free survival (DFS) were calculated from the date of ERH to the date of death, and local or distant recurrence, or last follow-up using the Kaplan–Meier method. Survival data were compared using the log-rank test. To identify factors associated with OS after ERH, the following clinicopathological parameters were analyzed: sex, age, body mass index (BMI), American Society of Anesthesiology (ASA) physical status, sequence of development of CRLM, comorbidities, Desmet score, alcohol or nicotine abuse, number and size of CRLM, Rat sarcoma viral oncogene (RAS) mutation, LiMAx, duration of surgery, number of administrated red blood cell (RBC) units, length of ICU and hospital stay, overall and liver-specific postoperative morbidity, revision surgery, readmission to ICU, and postoperative chemotherapy. Factors resulting in *p* < 0.100 in univariate analysis were then entered into a Cox regression analysis with backward elimination. *p* values lower than 0.05 were considered statistically significant. All statistical analyses were conducted using the SPSS software package, version 27 (IBM, Armonk, NY, USA) and R software (version 4.2.2, The R Foundation for Statistical Computing, Vienna, Austria).

## 3. Results

### 3.1. Baseline Characteristics

**Figure 1:**
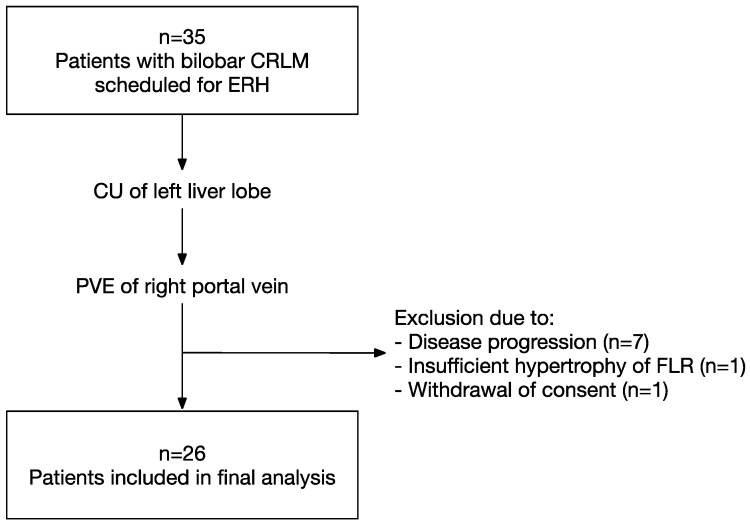
Flowchart of patients included in the study. Of 35 patients with bilobar CRLM who were eligible for study inclusion, 26 patients underwent CU, PVE and finally ERH. Nine patients (26%) were excluded because of tumor progression after CU and PVE (*n* = 7), insufficient hypertrophy of the FLR after PVE (*n* = 1) and withdrawal of consent for ERH (*n* = 1). CRLM, colorectal liver metastases; ERH, extended right hepatectomy; CU, clear-up; PVE, portal vein embolization; FLR, future liver remnant.

During the study period from 2015 to 2021, a total of 35 patients were identified to be scheduled for ERH for CRLM after CU of the left liver lobe followed by PVE. Of those, nine patients (26%) were unable to proceed to ERH due to disease progression (*n* = 7), insufficient hypertrophy of the FLR (*n* = 1), or withdrawal of consent to surgical treatment (*n* = 1), and referred to systemic treatment (see Figure 1). Consequently, the final analysis included 26 patients who successfully completed TSH. The median age at the time of ERH was 55 years (range: 34–77 years), and median BMI was 26 kg/m^2^ (range: 17–34 kg/m^2^). Arterial hypertension (35%) and pulmonary diseases (15%) were the most common comorbidities. Normal liver parenchyma or mild fibrosis (score 0–1 according to Desmet and Scheuer [26]) was seen in 91% of patients. All patients received chemotherapy prior to CU (Table 1). The median interval between PVE and ERH was five weeks (range: 4–11 weeks). Patients were then stratified by surgical approach of ERH in MIH (*n* = 12, 46%) and OH (*n* = 14, 54%). Clinical baseline characteristics were comparable between the groups (Table 1): No significant differences could be observed in terms of gender (*p* = 0.249), age (*p* = 0.940), BMI (*p* = 1), ASA physical status (*p* = 0.671), comorbidities, fibrosis score (*p* = 0.228), and the abuse of alcohol (*p* = 0.480) or nicotine (*p* = 1). Continuous data can be reviewed in Supplementary Figure S1.

### 3.2. Perioperative and Long-Term Outcomes

CU was performed via surgical resection, or a combination of resection and ablation in 83% and 8% for MIH, and 71% and 29% for OH, respectively (*p* = 0.652 and *p* = 0.330). One patient in the MIH group underwent ablation alone. Liver function, as measured by the LiMAx test, was comparable between the groups both before (295 vs. 296 µg/h/kg, *p* = 1) and after PVE (414 vs. 360 µg/h/kg, *p* = 0.131). Similarly, the calculated LiMAx of the FLR showed no significant difference before (80 vs. 90 µg/h/kg, *p* = 0.837) and after PVE (136 vs. 104 µg/h/kg, *p* = 0.063). For most patients, PVE was performed in a percutaneous transhepatic approach, while one patient in the OH group received a surgical transileocolic approach (*p* = 1). MIH procedures were performed hand-assisted laparoscopic, full laparoscopic and robotic-assisted in 8%, 25%, and 67% of cases, respectively. Three patients underwent trisectionectomy (*p* = 0.580). Duration of surgery was similar for MIH and OH (358 vs. 316 min, *p* = 0.705). Although the need for intraoperative red blood cell transfusions was lower during MIH, it did not reach statistical significance (17% vs. 50%, *p* = 0.110). The median length of ICU stay was one day in both groups (*p* = 0.899), but the median length of hospital stay was significantly shorter after MIH compared to OH (8 vs. 15 days, *p* = 0.008). Postoperative overall morbidity and major morbidity were 50% and 42% for MIH, and 79% and 64% for OH (*p* = 0.218 and *p* = 0.249). While the incidence of SSI tended to be lower after MIH (8% vs. 43%, *p* = 0.081), the incidences of intraabdominal abscess (*p* = 1), PHH (*p* = 1), biliary leakage (*p* = 1), and PHLF (*p* = 1) were equivalent between MIH and OH. Revision surgery was necessary in six cases (see Supplementary Table S2). No mortality was observed within 30 or 90 days after surgery in all patients (Table 2).

**Figure 2:**
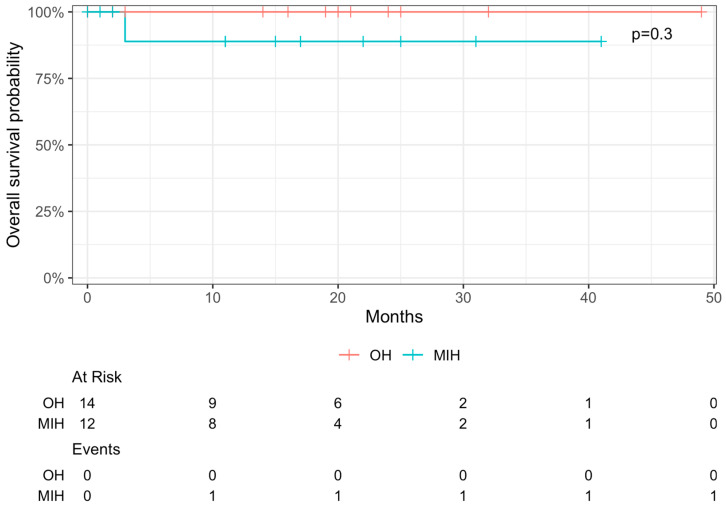
Overall survival of 26 patients who underwent TSH for extended CRLM, stratified by approach in MIH and OH. Median follow-up was 16 months. Three-year overall survival was 89% and 100% for MIH and OH, respectively (*p* = 0.3).

**Figure 3:**
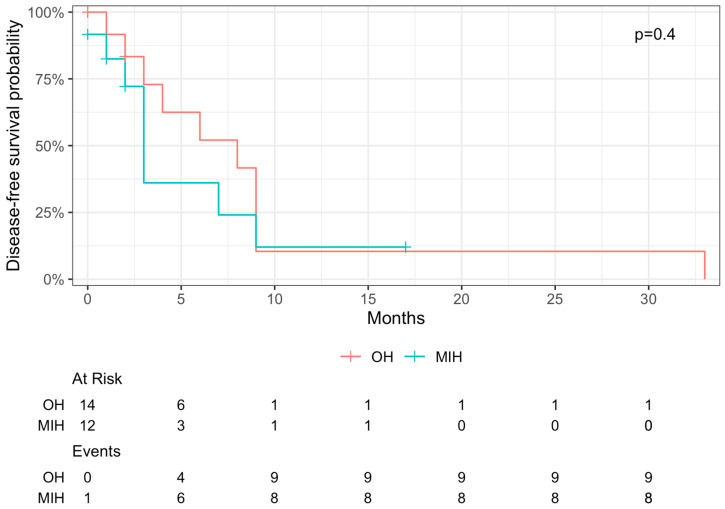
Disease-free survival of 26 patients who underwent TSH for extended CRLM, stratified by approach in MIH and OH. One-year disease-free survival was 12% and 10% for MIH and OH, respectively (*p* = 0.4).

After a median follow-up of 16 months, three-year overall survival was 89% for MIH and 100% for OH (*p* = 0.292; Figure 2). One-year disease-free survival was 12% for MIH and 10% for OH (*p* = 0.416; Figure 3). OS comparison between patients after ERH and patients who dropped out after CU and PVE can be found in Supplementary Figure S2.

Results of univariate and multivariate analysis of factors associated with OS are summarized in Supplementary Table S1. As indicated in univariate analysis, OS was significantly influenced by age at resection (*p* = 0.021), the calculated LiMAx of the FLR after PVE (*p* = 0.094), and the development of postoperative biliary leakage (*p* = 0.094). However, multivariate analysis failed to identify factors that were independently associated with worse OS.

### 3.3. Type of Recurrence and Therapy of Recurrence

Recurrent disease occurred in 67% of patients after MIH and 71% after OH (*p* = 1; Table 3). Hepatic recurrence was observed in four cases in both groups (*p* = 1). Recurrence at other sites or multiple sites including hepatic recurrence were evident in three and one cases for MIH, and four and two cases for OH (*p* = 1 and *p* = 1). Recurrence was accessible to surgical (33%) or local ablative therapy (11%), while recurrent disease was treated by systemic chemotherapy alone in 44% of cases.

## 4. Discussion

In our case series, we evaluated the concept of ERH within a TSH protocol including CU and PVE for patients with extensive bilobar CRLM. Our findings revealed comparable postoperative short- and long-term outcomes after MIH and OH. Notably, the length of stay was reduced after MIH. These results indicate that TSH with ERH for extended CRLM is surgically feasible and safe in both open and minimally invasive settings. Furthermore, multidisciplinary treatment including extended surgery achieved a three-year OS rate of 95%, suggesting that this approach may offer long-term survival benefits for carefully selected patients.

While the indications for surgical treatment of extended CRLM continue to expand, a significant proportion of patients remain ineligible for liver resection, which currently still offers the most effective treatment option for long-term survival [32,33]. Therefore, expanding the pool of patients suitable for surgical resection is a crucial aspect of the multidisciplinary therapeutic approach. Advances in systemic chemotherapy have allowed for more patients with extensive CLRM to undergo liver resection through down-sizing chemotherapy strategies. Moreover, staged procedures with FLR augmentation through PVE have further increased resectablity rates [34,35,36,37,38,39,40,41,42]. In this study, we specifically evaluated the concept of TSH with PVE after CU of the left liver lobe. Previous studies have demonstrated the feasibility of this approach and reported favorable short- and long-term outcomes after standard and extended hepatectomies [34,43,44]. However, we focused on extended liver resections in this study to minimize heterogeneity, and, to the best of our knowledge, this study is the first to exclusively evaluate ERH in this setting. Although a recent French study included 30% extended hepatectomies, a subgroup analysis was not reported [35]. Furthermore, we limited the clinical analyses to the second stage of TSH, as this procedure is technically more demanding and carries greater risks, and therefore represents the critical part of TSH.

After ERH, we observed a major morbidity rate of 54% without any 90-day postoperative mortality, which is consistent with previous studies reporting morbidity rates ranging from 10–59% and mortality rates from 0–7% [9,11,34,35,44,45]. However, these studies included both extended and standard right hepatectomies within the setting of TSH. More specifically, postoperative rates of PHH, PHLF, and biliary leakage were mainly limited to grade A and B in our study. Revision surgery and readmission to ITS were required in only six and four cases, respectively. When comparing MIH and OH, postoperative morbidity rates were comparable between both approaches. However, the MIH group had a significantly shorter hospital stay and a reduced need for intraoperative transfusions. Although previous research has shown that blood loss predicts morbidity and mortality after hepatic resection [46,47], our results did not demonstrate a significant difference between the groups. Interestingly, despite all patients having a history of prior abdominal surgery after CU, there was no need for conversion to open surgery in the MIH group. This conversion rate is lower than reported data from two other studies comparing (extended) hepatectomy in a similar setting, reporting conversion rates of 11–15% [34,35]. These data support recent evidence that minimally invasive approaches are feasible even in patients with a history of abdominal surgery [22]. In summary, our findings align with the results of recent studies comparing laparoscopic and open TSH [34,35,45], indicating that both approaches can be safely performed with comparable short-term outcomes. In addition, our results may support the recommendation from the Southhampton Consensus guidelines on laparoscopic liver surgery (LLS) for a stepwise implementation of LLS in specialist liver centers, which may be expanded for ERH after CU based on our results [48].

An alternative surgical strategy for bilobar CRLM is ALPPS, which was originally introduced to allow for a more rapid hepatic hypertrophy than PVE [49]. Since then, several modifications of ALPPS have been reported including CU of the liver remnant for extensive CRLM during the performance of ALPPS [50,51,52]. However, only few case series exist and none of them included exclusively patients with CRLM who underwent ALPPS with CU, making a comparison with our results difficult [53,54]. Generally, though ALPPS was associated with a greater increase of the FLR and more frequent completion of the second stage of TSH, it has a tendency to higher postoperative morbidity and mortality than TSH with PVE [55]. Considering this, TSH with PVE is the preferred strategy for extensive bilobar CLRM in our institution.

Our study reported a three-year OS rate of 95%, which is one of the highest compared to existing data [9]. While long-term survival is clearly limited by recurrence, surgical resection margins and the management of recurrent disease are crucial factors affecting OS. Surgery achieved a R0-resection in 76% of patients, which is in accordance with the results of a recent meta-analysis reporting a R0-resection rate of 75% [9]. Interestingly, the occurrence of local recurrence after R1 resection was low, supporting previous findings that positive resection margins may serve as a surrogate parameter for advanced disease without influencing the location of recurrence, as it is associated with an increased risk not only for local recurrence in general but also distant metastatic disease [56,57].

Still, DFS after one year was only 12% and 10% after MIH and OH, respectively, which is lower than a reported median DFS of 20% in the literature [9]. As we only included patients who underwent extensive liver resection with ERH, we believe that these low DFS numbers may be caused by a higher tumor burden in our cohort. Nevertheless, we were able to achieve a three-year OS of 95%, which may mainly be attributed to our management of recurrent disease. The preferred treatment approach for recurrent disease is repeated resection at our institution, which was performed in 23% of affected patients. The high percentage of patients undergoing repeated hepatectomy may have contributed to the favorable OS reported in our series [58,59,60,61]. A recent study by Takahashi et al. reported an 87% recurrence rate, but favorable long-term survival was achieved through repeated resection [62]. Conversely, data on systemic therapy for recurrent CRLM have shown median survival rates of 11–29 months and a five-year OS of 20% [63,64,65]. Multivariate analysis in our study did not reveal parameters independently associated with worse OS. Nevertheless, univariate analysis identified advanced age, inadequate preoperative LiMAx value, and the occurrence of postoperative bile leakage as factors linked to worse OS. These findings underscore the critical role of meticulous patient selection for TSH. Although elderly patients have often been associated with unfavorable postoperative outcomes in abdominal surgery [66,67,68,69], the recent literature suggests that liver resection can be considered even for patients with advanced age following thorough preoperative evaluation [70]. Moreover, our analysis highlights that achieving a sufficient FLR with optimal liver function, as assessed by the LiMAx test, reduces the risk of compromised OS due to PHLF, ultimately safeguarding patients from associated morbidity and mortality [71,72]. In the future, computational models may further assist in decreasing the probability of PHLF [73,74]. Lastly, our data support the notion that postoperative bile leakage is a known factor impacting long-term outcomes for patients after liver surgery [75].

The use of interval chemotherapy during the liver growth period after PVE, or after CU, remains a topic of debate. On the one hand, the non-embolized liver parenchyma requires time for regeneration and growth, as studies on FLR hypertrophy kinetics after PVE have demonstrated that the maximum volume increase occurs within the first three weeks after PVE [76]. On the other hand, tumor progression during the interval between the two stages of TSH may lead to unresectability, and clinical data suggest that PVE might even accelerate tumor progression [77,78,79,80]. In our study, we report a drop-out rate of 26% of patients, who did not proceed to the second step of TSH. Among these patients, tumor progression was indeed the most common reason for drop-out. Previous studies investigating the drop-out rate between the two stages have reported similar rates ranging from 24 to 38% [9,81,82]. Interval chemotherapy was not administered in this study, as previous research has suggested a negative impact on liver parenchyma regeneration [83,84]. Furthermore, Muratore et al. found that interval chemotherapy did not reduce the drop-out rate between the two stages [85]. In contrast, other studies have indicated that chemotherapy does not impair liver regeneration [86,87,88]. Goere et al. even reported no significant difference in the rate of hypertrophy after PVE whether chemotherapy was interrupted one month before PVE or continued until surgery [87]. In our study, the interval between PVE and ERH was five weeks, which is relatively short compared to other studies reporting intervals of 4–11 weeks [11,34,35,43,44]. A shorter chemotherapy-free interval may improve oncological outcomes and could be associated with improved OS, as observed in our study [89]. However, the chemotherapy-free interval itself, the fact that we induced liver hypertrophy through PVE, and careful patient selection may have contributed to our low rate of PHLF [83,84]. While comparable studies have reported PHLF rates between 5 and 8% [34,35], we did not observe any cases of grade B or grade C liver failure, despite all patients receiving prior chemotherapy and undergoing extended liver resection. In addition, PVE has been known to reduce the incidence of PHLF by augmenting the FLR, thus enhancing the safety of hepatectomy. In a prospective trial examining PVE before major hepatectomy, Farges et al. reported PHLF rates of 7% and 50% with and without preoperative PVE, respectively [90]. Other studies reported similar rates of 4–8% for PHLF following major hepatectomy after PVE [90,91,92,93].

In conclusion, our study establishes the feasibility and safety of the TSH approach, particularly in the context of extended hepatectomies. These findings provide valuable insights for devising a treatment plan tailored to patients with extensive bilobar CRLM. When assessing these patients for CRLM surgery, we recommend incorporating the following factors into consideration: patient-centered parameters, including general health status and comorbidities, tumor biology (including mutational characteristics and the effectiveness of applied chemotherapy), technical resectability (considering the relationship to anatomical structures such as blood vessels or bile ducts), and functional resectability (ensuring adequate liver function of the future liver remnant after surgery).

Finally, there are certain limitations of our study. Due to the retrospective study design and rather small cohort, conclusions from the results have to be interpreted with caution. The diversity of procedures for surgical treatment of bilobar CRLM poses a challenge in study design and comparison across different series. To address this issue, we focused on ERH for CRLM within the setting of TSH. The extent of liver resection is a known predictor for morbidity and mortality, which are increased in case of extended hepatectomies [46,94,95,96,97,98], this should be considered when comparing our results to existing studies.

## 5. Conclusions

Our study demonstrated that ERH in combination with clearance of the left liver lobe and PVE for CRLM can be conducted with favorable short- and long-term outcomes for carefully selected patients at specialized centers. The adoption of minimally invasive techniques may help to reduce hospital stay and the need for blood transfusions. Regarding long-term outcomes, minimally invasive and open approaches proved to be equivalent. Further prospective studies are needed to corroborate our results, as conclusions from this single-center case series should be carefully drawn.

## Figures and Tables

**Table 1:** Clinicopathological data of 26 patients who underwent TSH for extended CRLM, stratified by approach in MIH and OH.

Variable	All Cases (*n* = 26)	MIH (*n* = 12)	OH (*n* = 14)	*p*
Gender, *n* (%)				0.249
Female	12 (46)	7 (58)	5 (36)	
Male	14 (54)	5 (42)	9 (64)	
Age, years, median (range)	55 (34–77)	58 (34–71)	50 (40–77)	0.940
Age > 65 years, *n* (%)	5 (19)	1 (8)	4 (29)	0.330
BMI, kg/m^2^, median (range)	25 (17–34)	25 (19–33)	26 (17–34)	1
BMI > 30 kg/m^2^, *n* (%)	5 (19)	3 (25)	2 (14)	0.635
ASA physical status, *n* (%)				0.671
II	12 (46)	5 (42)	7 (50)	
III	14 (54)	7 (58)	7 (50)	
Timing of metastasis, *n* (%)				0.225
synchronous	23 (89)	12 (100)	11 (79)	
metachronous	3 (12)	0 (0)	3 (21)	
Comorbidities, *n* (%)				
Diabetes	1 (4)	0 (0)	1 (7)	1
Hypertension	9 (35)	5 (42)	4 (29)	0.683
Coronary heart disease	0 (0)	0 (0)	0 (0)	-
Pulmonary disease	4 (15)	3 (25)	1 (7)	0.306
Renal disease	0 (0)	0 (0)	0 (0)	-
Liver fibrosis/cirrhosis (Desmet/Scheuer), *n* (%)				0.228
none	12 (55)	7 (64)	5 (46)	
Grade 1	8 (36)	2 (18)	6 (55)	
Grade 2	1 (5)	1 (9)	0 (0)	
Grade 3	1 (5)	1 (9)	0 (0)	
Grade 4	0 (0)	0 (0)	0 (0)	
Desmet-Score ≥ 3, *n* (%)	1 (5)	1 (9)	0 (0)	1
Alcohol abuse, *n* (%)	1 (4)	1 (8)	0 (0)	0.480
Nicotine abuse, *n* (%)	3 (12)	1 (8)	2 (15)	0.588
Previous abdominal surgery, *n* (%)	26 (100)	12 (100)	14 (100)	-
Preoperative chemotherapy, *n* (%)	26 (100)	12 (100)	14 (100)	-
Number of CRLM after CU, median (range)	6 (2–19)	7 (2–19)	6 (3–14)	0.297
Size of biggest CRLM after CU, mm, median (range)	37 (12–130)	37 (12–65)	39 (14–130)	0.560
RAS mutation, *n* (%)	8 (33)	2 (17)	6 (59)	0.193
BRAF mutation, *n* (%)	0 (0)	0 (0)	0 (0)	-
MSI, *n* (%)	0 (0)	0 (0)	0 (0)	-

MIH, minimally invasive hepatectomy; OH, open hepatectomy; BMI, body mass index; ASA, American Society of Anesthesiology; CRLM, colorectal liver metastasis; CU, clear-up; RAS, Rat sarcoma viral oncogene; BRAF, v-Raf murine sarcoma viral oncogene homolog B; MSI, microsatellite instability.

**Table 2:** Perioperative and oncological data of 26 patients who underwent TSH for extended CRLM, stratified by approach in MIH and OH.

Variable	All Cases (*n* = 26)	MIH (*n* = 12)	OH (*n* = 14)	*p*
Type of CU, *n* (%)				
Resection	20 (77)	10 (83)	10 (71)	0.652
Ablation	1 (4)	1 (8)	0 (0)	0.462
Combination	5 (19)	1 (8)	4 (29)	0.330
Simultaneous resection of primary tumor during CU, *n* (%)	9 (35)	3 (25)	6 (43)	0.429
LiMAx before PVE, µg/kg/h, median (range)	296 (195–537)	295 (206–517)	296 (195–537)	1
Calculated FLR-LiMAx before PVE, µg/kg/h, median (range)	85 (54–159)	80 (54–159)	90 (54–111)	0.837
LiMAx after PVE, µg/kg/h, median (range)	374 (151–659)	414 (151–545)	360 (182–659)	0.131
Calculated FLR-LiMAx after PVE, µg/kg/h, median (range)	120 (78–240)	136 (78–240)	104 (85–185)	0.063
Approach of PVE, *n* (%)				1
Percutaneous transhepatic	25 (96)	12 (100)	13 (93)	
Surgical transileocolic	1 (4)	0 (0)	1 (7)	
Type of MIH, *n* (%)				
Laparoscopic surgery	-	2 (25)	-	-
HALS	-	1 (8)	-	-
Robotic-assisted surgery	-	8 (67)	-	-
Extent of hepatectomy, *n* (%)				0.580
Trisectionectomy	3 (12)	2 (17)	1 (7)	
Extended right hepatectomy	23 (89)	10 (83)	13 (93)	
Duration of surgery, minutes, median (range)	334 (197–605)	358 (207–605)	316 (197–483)	0.705
Need for intraoperative RBC transfusion, *n* (%)	9 (35)	2 (17)	7 (50)	0.110
Number of RBCs, median (range)	0 (0–5)	0 (0–2)	1 (0–5)	0.145
Positive resection margins, *n* (%)	6 (24)	2 (17)	4 (31)	0.645
Length of ICU stay, days, median (range)	1 (1–91)	1 (1–7)	1 (1–91)	0.899
Length of hospital stay, days, median (range)	12 (5–98)	8 (5–39)	15 (8–98)	0.008
SSI, *n* (%)	7 (27)	1 (8)	6 (43)	0.081
Abscess, *n* (%)	8 (31)	4 (33)	4 (29)	1
PHH, *n* (%)				1
Grade A	3 (12)	1 (8)	2 (14)	
Grade B	0 (0)	0 (0)	0 (0)	
Grade C	0 (0)	0 (0)	0 (0)	
All categories	3 (12)	1 (8)	2 (14)	1
Biliary leakage, *n* (%)				0.636
Grade A	0 (0)	0 (0)	0 (0)	
Grade B	6 (23)	3 (25)	3 (21)	
Grade C	1 (4)	0 (0)	1 (7)	
All categories	7 (27)	3 (25)	4 (29)	1
PHLF, *n* (%)				1
Grade A	3 (12)	1 (8)	2 (14)	
Grade B	0 (0)	0 (0)	0 (0)	
Grade C	0 (0)	0 (0)	0 (0)	
All categories	3 (12)	1 (8)	2 (14)	1
Postoperative dialysis, *n* (%)	1 (4)	0 (0)	1 (7)	1
Revision surgery, *n* (%)	6 (23)	1 (8)	5 (36)	0.170
Readmission to ITS, *n* (%)	4 (15)	1 (8)	3 (21)	0.598
Clavien-Dindo, *n* (%)				0.374
1	2 (8)	0 (0)	2 (14)	
2	1 (4)	1 (8)	0 (0)	
3a	7 (27)	3 (25)	4 (29)	
3b	4 (15)	1 (8)	3 (21)	
4	3 (12)	1 (8)	2 (14)	
5	0 (0)	0 (0)	0 (0)	
Postoperative morbidity, *n* (%)	17 (65)	6 (50)	11 (79)	0.218
Postoperative major morbidity, *n* (%)	14 (54)	5 (42)	9 (64)	0.249
30-day mortality, *n* (%)	0 (0)	0 (0)	0 (0)	-
90-day mortality, *n* (%)	0 (0)	0 (0)	0 (0)	-
Postoperative chemotherapy, *n* (%)	11 (42)	4 (33)	7 (50)	0.684

MIH, minimally invasive hepatectomy; OH, open hepatectomy; CU, clear up; LiMAx, liver Maxi-mum capacity test; PVE, portal vein embolization; FLR, future liver remnant; HALS, hand-assisted laparoscopic surgery; RBC, red blood cell; ICU, intensive care unit; SSI, surgical site infection; PHH, post-hepatectomy hemorrhage; PHLF, post-hepatectomy liver failure.

**Table 3:** Recurrent disease and respectable treatment in 26 patients who underwent TSH for extended CRLM, stratified by approach in MIH and OH.

Variable	All Cases (*n* = 26)	MIH (*n* = 12)	OH (*n* = 14)	*p*
Recurrence, *n* (%)	18 (69)	8 (67)	10 (71)	1
Hepatic	8 (44)	4 (50)	4 (40)	1
Other localization than hepatic	7 (39)	3 (38)	4 (40)	1
Combination	3 (17)	1 (12)	2 (20)	1
Therapy of recurrence, *n* (%)				
Surgical	6 (33)	3 (38)	3 (30)	1
Local ablative	2 (11)	1 (12)	1 (10)	1
Systemic chemotherapy	8 (44)	4 (50)	4 (40)	1
No therapy or lost to follow-up	2 (11)	0 (0)	2 (20)	1

MIH, minimally invasive hepatectomy; OH, open hepatectomy.

## Data Availability

The data presented in this study are available on reasonable request from the corresponding author. The data are not publicly available due to sensitive patient information.

## Supplementary Materials

The following supporting information can be downloaded at: https://www.mdpi.com/article/10.3390/curroncol31030085/s1, Figure S1: Boxplots of continuous data showing group comparison between MIH and OH. (A) Age at resection, in years; (B) BMI, in kg/m^2^; (C) number of CRLM after CU; (D) size of biggest CRLM, in mm; (E) LiMAx before PVE, in µg/kg/h; (F) calculated LiMAx of FLR before PVE, in µg/kg/h; (G) LiMAx after PVE, in µg/kg/h; (H) calculated LiMAx of FLR after PVE, in µg/kg/h; (I) duration of surgery, in minutes; (J) number of intraoperative RBC units; (K) length of ICU stay, in days; (L) length of hospital stay, in days. Boxes showing minimum to maximum, line at median. MIH, minimally invasive hepatectomy; OH, open hepatectomy; BMI, body-mass index; CRLM, colorectal liver metastases; CU, clear-up; PVE, portal vein embolization; FLR, future liver remnant; RBC, red blood cells; ICU, intensive care unit; NS, not significant; **, *p* < 0.010; Figure S2: Overall survival (OS) of all 35 patients who were eligible for this study, and who underwent clear-up (CU) of the left liver lobe followed by embolization of the right portal vein (PVE). Afterwards, nine patients (26%) dropped out due to disease progression, insufficient hypertrophy of the future liver remnant (FLR) after PVE, or withdrawal of consent for surgery, and were recommended for systemic treatment. In this survival analysis, OS between patients who did not proceed to ERH after CU (“Dropout”) were compared to those who underwent ERH. OS was calculated from the day of CU until the day of death or last follow-up. One-year OS rates were 100% and 95% in the Dropout and ERH groups, respectively (*p* = 0.3); Table S1: Univariate and multivariate analysis of factors associated with overall and disease-free survival in 26 patients who underwent TSH for extended CRLM; Table S2: Indications for revision surgery, which was necessary in six cases (23%).

## Abbreviations and Acronyms

ALPPS	associating liver partition with portal vein ligation for staged hepatectomy
ASA	American Society of Anesthesiology
BMI	Body mass index
CDC	Centers for Disease Control and Prevention
CRC	colorectal cancer
CRLM	colorectal liver metastases
CU	clear-up
DFS	disease-free survival
ERH	extended right hepatectomy
FLR	future liver remnant
HALS	hand-assisted laparoscopic surgery
ICU	intensive care unit
ISGLS	International Study Group of Liver Surgery
LiMAx	maximum liver function capacity
LLS	laparoscopic liver surgery
MIH	minimally invasive hepatectomy
MSI	microsatellite instability
OH	open hepatectomy
OS	overall survival
PHH	post-hepatectomy hemorrhage
PHLF	post-hepatectomy liver failure
PVE	portal vein embolization
RAS	Rat sarcoma viral oncogene
RBC	red blood cell
RS	robotic surgery
SSI	surgical site infections
TSH	two-stage hepatectomy
