# Supplementary material for: Extended Right Hepatectomy following Clearance of the Left Liver Lobe and Portal Vein Embolization for Curatively Intended Treatment of Extensive Bilobar Colorectal Liver Metastases: A Single-Center Case Series
Source: Curr Oncol. 2024 Feb 21;31(3):1145–61. doi: 10.3390/curroncol31030085 (PMC10969123; doi:10.3390/curroncol31030085)
Supplement: Supplementary file 1 [file curroncol-31-00085-s001.zip › curroncol-2798007-supplementary.pdf]

Supplementary Data and Figures

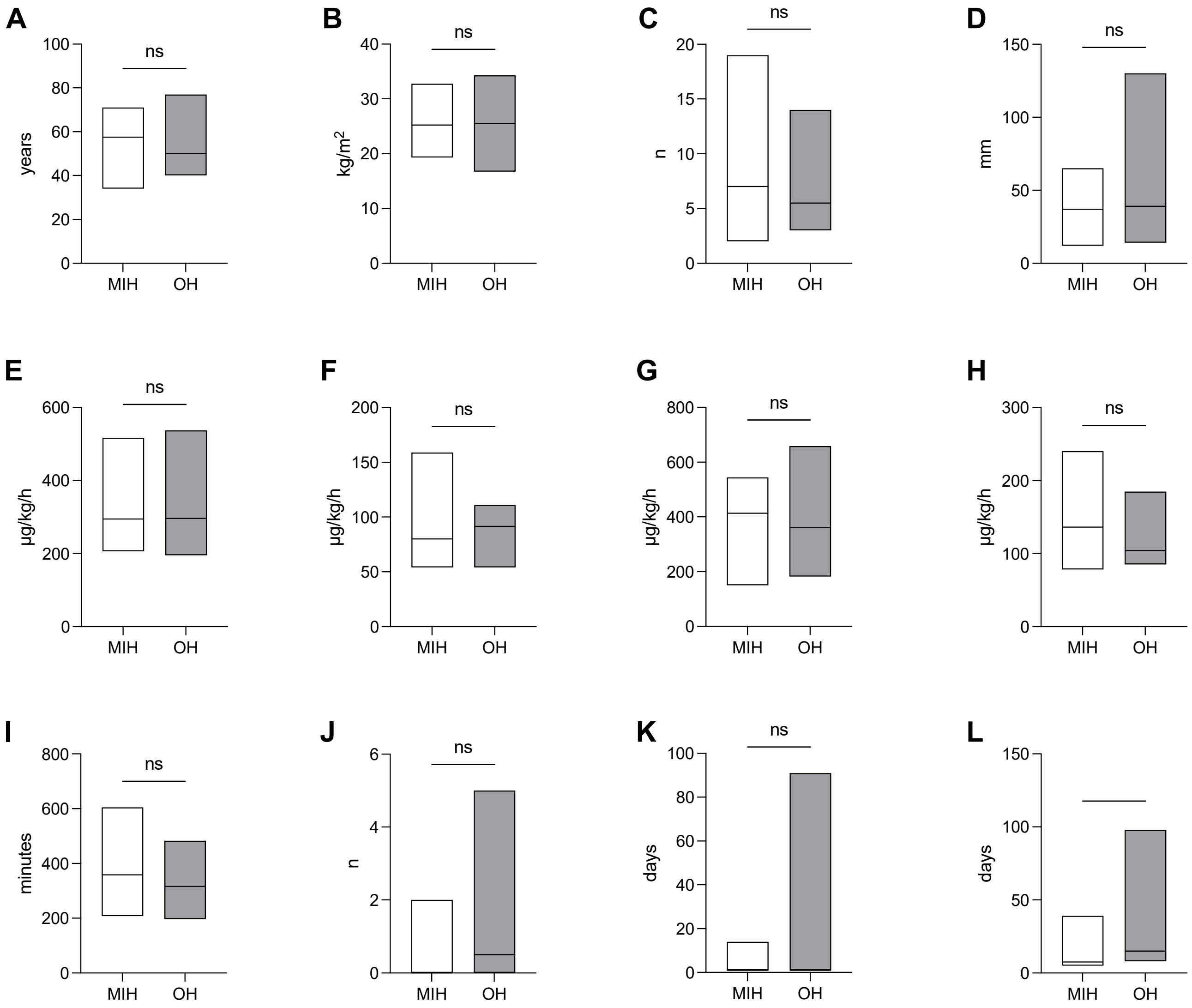

Supplementary Figure S1. Boxplots of continuous data showing group comparison between MIH and OH. A) Age at resection, in years; B) BMI, in kg/m^2; C) number of CRLM after CU; D) size of biggest CRLM, in mm; E) LiMAx before PVE, in µg/kg/h; F) calculated LiMAx of FLR before PVE, in µg/kg/h; G) LiMAx after PVE, in µg/kg/h; H) calculated LiMAx of FLR after PVE, in µg/kg/h; I) duration of surgery, in minutes; J) number of intraoperative RBC units; K) length of ICU stay, in days; L) length of hospital stay, in days. Boxes showing minimum to maximum, line at median. MIH, minimally invasive hepatectomy; OH, open hepatectomy; BMI, body-mass index; CRLM, colorectal liver metastases; CU, clear-up; PVE, portal vein embolization; FLR, future liver remnant; RBC, red blood cells; ICU, intensive care unit; NS, not significant; **, $p < 0.010$.

Supplementary Table S1. Univariate and multivariate analysis of factors associated with overall and disease-free survival in 26 patients who underwent TSH for extended CRLM.

**Predictor of survival**	**n (%)**	**Overall survival**		
		**UV**	**MV†**	
		P	***P***	**HR (95% CI)**
Male sex	14 (54)	0.343		

Age >65 years	5 (19)	**0.021**	NS	-
BMI >30 kg/m²	5 (19)	0.665		
ASA ≥III	14 (54)	0.394		
Synchronous CRLM	23 (89)	-		
Diabetes	1 (4)	-		
Hypertension	9 (35)	0.141		
Pulmonary disease	4 (15)	0.606		
Desmet-Score ≥3	1 (5)	0.803		
Alcohol abuse	1 (4)	0.814		
Nicotine abuse	3 (12)	0.732		
Number of CRLM ≥6	14 (54)	0.343		
Size of biggest CRLM ≥37 mm	13 (50)	0.292		
RAS mutation	8 (33)	0.121		
Calculated FLR-LiMAx post-PVE ≤100 µg/kg/h	6 (23)	**0.094**	NS	-
MIH	12 (46)	0.292		
Duration of surgery ≥334 minutes	13 (50)	0.394		
Number of RBC units ≥1	9 (35)	0.445		
Length of ICU stay >1 day	10 (39)	0.190		
Length of hospital stay ≥12 days	13 (50)	0.343		
SSI	7 (27)	0.550		
Abscess	8 (31)	0.141		
PHH, all grades	3 (12)	0.814		
Biliary leakage, all grades	7 (27)	**0.094**	NS	-
PHLF, all grades	3 (12)	0.732		
Postoperative dialysis	1 (4)	0.814		
Revision surgery needed	6 (23)	0.606		
Readmission to ICU	4 (15)	0.732		
Postoperative morbidity	17 (65)	0.445		
Postoperative major morbidity	14 (54)	0.343		
Postoperative chemotherapy	11 (42)	0.503		

† Cox regression multivariate analysis included all variables with p < 0.100 in univariate analysis.
UV, univariate analysis; MV, multivariate analysis; BMI, body-mass index; ASA, American Society of Anesthesiology; CRLM, colorectal liver metastases; RAS, Rat sarcoma viral oncogene; FLR, future liver remnant; PVE, portal vein embolization; MIH, minimally invasive hepatectomy; RBC, redd blood cell; SSI, surgical site infection; PHH, post-hepatectomy hemorrhage; PHLF, post hepatectomy liver failure; ICU, intensive care unit; HR, hazard ratio; CI, confidence interval; NS, not significant

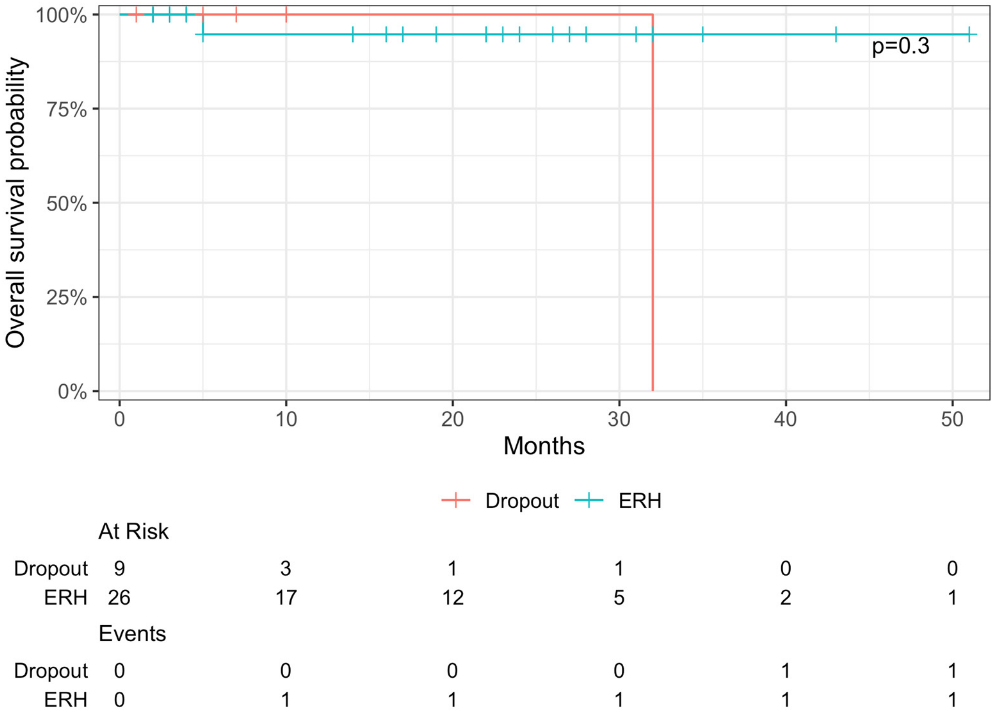

Supplementary Figure S2. Overall survival (OS) of all 35 patients who were eligible for this study, and who underwent clear-up (CU) of the left liver lobe followed by embolization of the right portal vein (PVE). Afterwards, nine patients (26%) dropped out due to disease progression, insufficient hypertrophy of the future liver remnant (FLR) after PVE, or withdrawal of consent for surgery, and were recommended for systemic treatment. In this survival analysis, OS between patients who did not proceed to ERH after CU (“Dropout”) were compared to those who underwent ERH. OS was calculated from the day of CU until the day of death or last follow-up. One-year OS rates were 100% and 95% in the Dropout and ERH groups, respectively (p = 0.3).

Supplementary Table S2. Indications for revision surgery, which was necessary in six cases (23%).

**Indications for revision surgery**
Placement of drainages for necrotic pancreatitis
Stopping bleeding and removal of hematoma
Debridement for wound healing disturbance
Removal of pleural empyema because of bilio-pleural fistula
Small bowel perforation
Fascial dehiscence
